# Supplementary material for: Comprehensive Phylogenomics of Methylobacterium Reveals Four Evolutionary Distinct Groups and Underappreciated Phyllosphere Diversity
Source: Genome Biol Evol. 2022 Jul 30;14(8):evac123. doi: 10.1093/gbe/evac123 (PMC9364378; doi:10.1093/gbe/evac123)

Figure S1 : Proportion of genes per genome present in 1 (blue), 2 (green), 3 (orange), 4 (red) or 5 (purple) copies per genome in function of genome assembly quality (given by the number of scaffolds per genome, log scale).

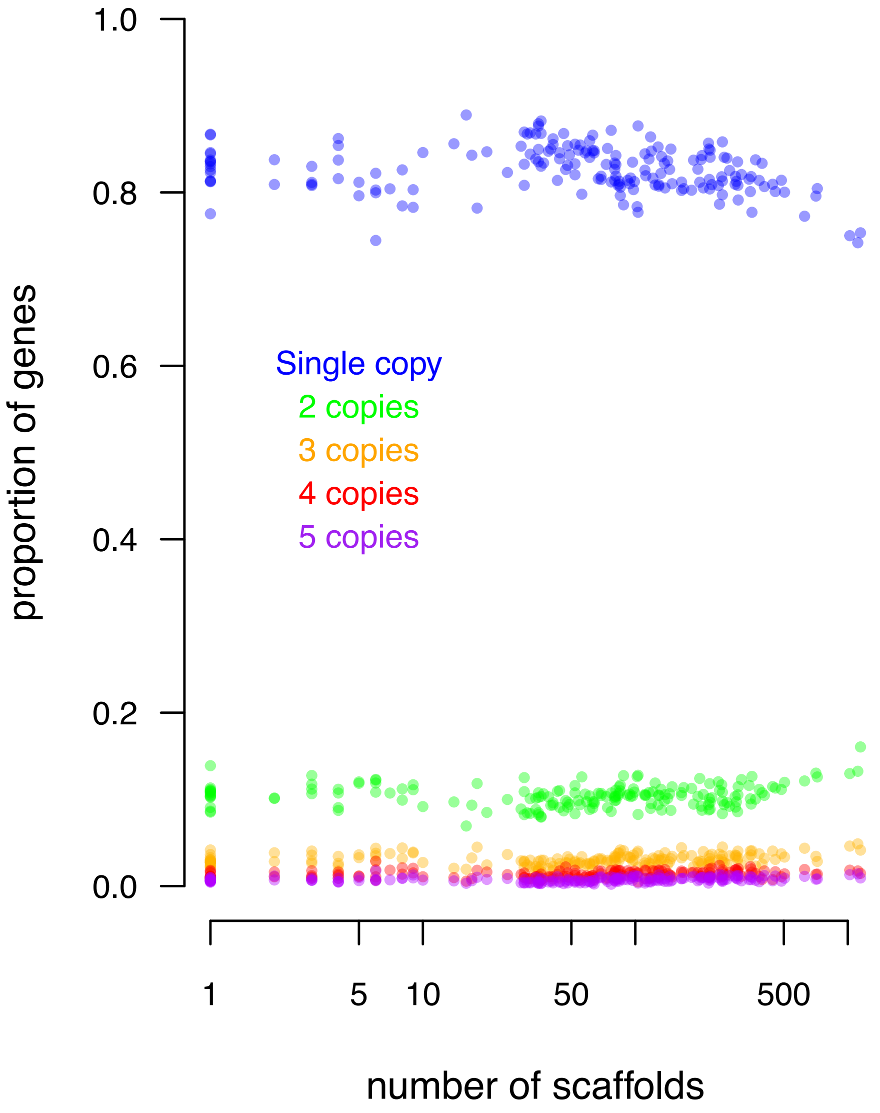

Supplement: evac123_Supplementary_Data [file evac123_supplementary_data.zip › Figure-S1.pdf]
