# Supplementary material for: Comprehensive Phylogenomics of Methylobacterium Reveals Four Evolutionary Distinct Groups and Underappreciated Phyllosphere Diversity
Source: Genome Biol Evol. 2022 Jul 30;14(8):evac123. doi: 10.1093/gbe/evac123 (PMC9364378; doi:10.1093/gbe/evac123)

Figure S4: Normalized RF distance distribution between the RAxML majority consensus rule lineage tree and the 512 replicate trees (grey; see Figure 1a) and normalized RF distances between lineage trees (points; legend on top right).

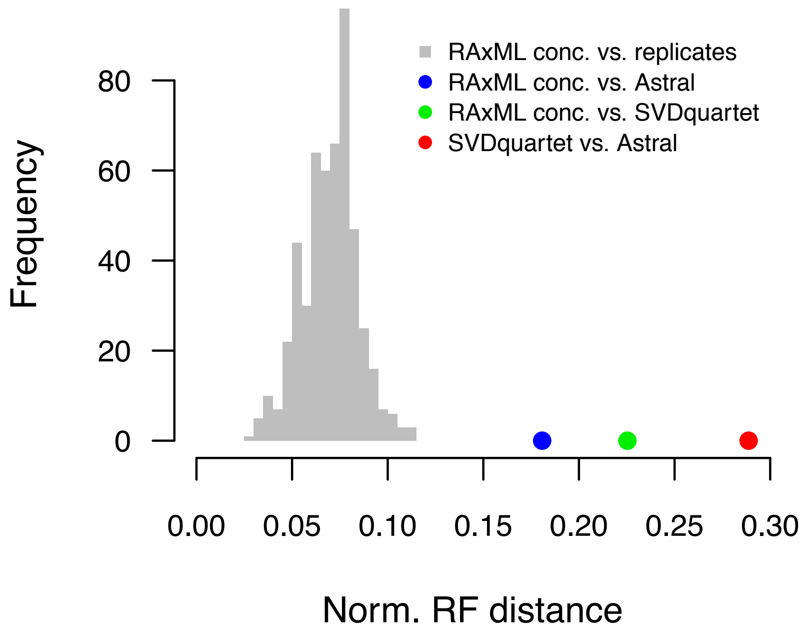

Supplement: evac123_Supplementary_Data [file evac123_supplementary_data.zip › Figure-S4.pdf]
