# Supplementary material for: Comprehensive Phylogenomics of Methylobacterium Reveals Four Evolutionary Distinct Groups and Underappreciated Phyllosphere Diversity
Source: Genome Biol Evol. 2022 Jul 30;14(8):evac123. doi: 10.1093/gbe/evac123 (PMC9364378; doi:10.1093/gbe/evac123)

Figure S7: Pan (a) and core (b) genome size estimations in four *Methylobacterium* groups and *Microvirga*. Genome sizes per group (number of genes per groups; y-axis) were estimated for every number of species assumed in the range 1-n (n = maximum number of species per group; x-axis). For each group and number of species, average (lines) and standard deviation (frames) over 100 random resampling of n species per group were estimated. Dotted lines indicate the value for which pan genome and core genome size where estimated (n=15 species per group; Figure 2).

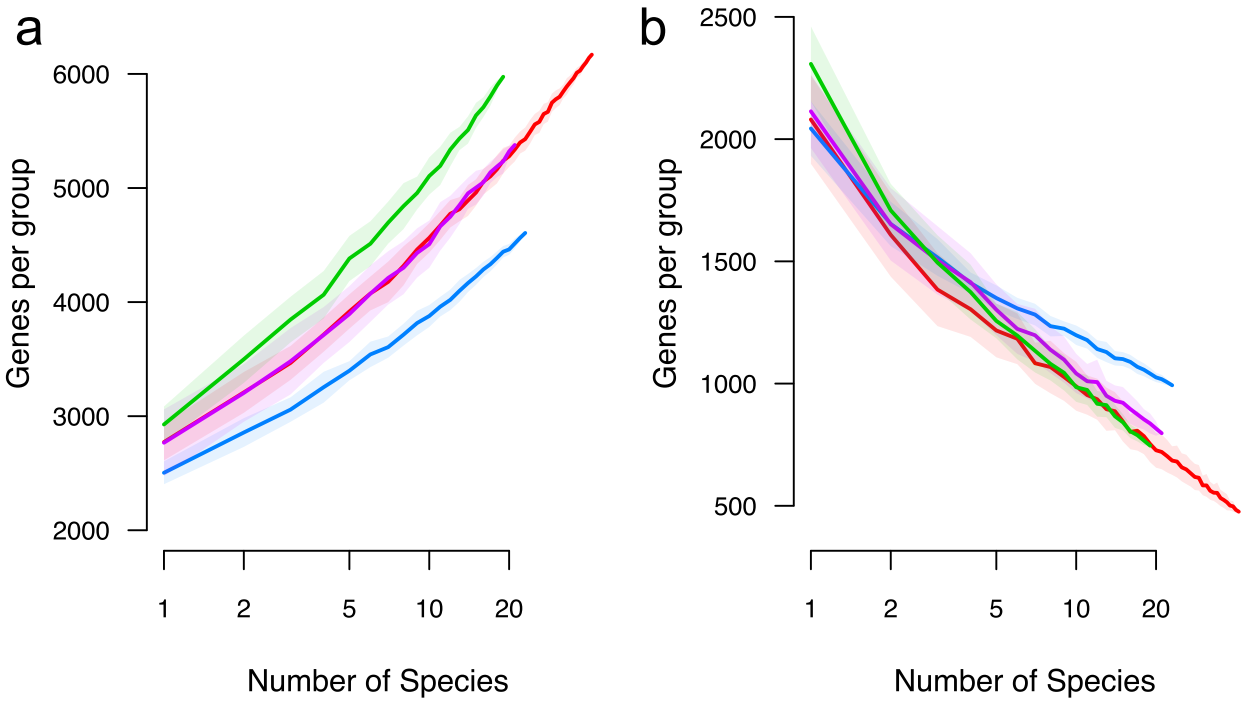

Supplement: evac123_Supplementary_Data [file evac123_supplementary_data.zip › Figure-S7-New.pdf]
