# Supplementary material for: Comprehensive Phylogenomics of Methylobacterium Reveals Four Evolutionary Distinct Groups and Underappreciated Phyllosphere Diversity
Source: Genome Biol Evol. 2022 Jul 30;14(8):evac123. doi: 10.1093/gbe/evac123 (PMC9364378; doi:10.1093/gbe/evac123)

Figure S8: a) ASTRAL species tree. b) Heatmap of dissimilarity in gene content (*BC* index; blue scale; above diagonal) and similarity in core genome architecture (Synteny index; orange scale; below diagonal) among 104 *Methylobacterium* and 20 outgroups species, matching the species tree.

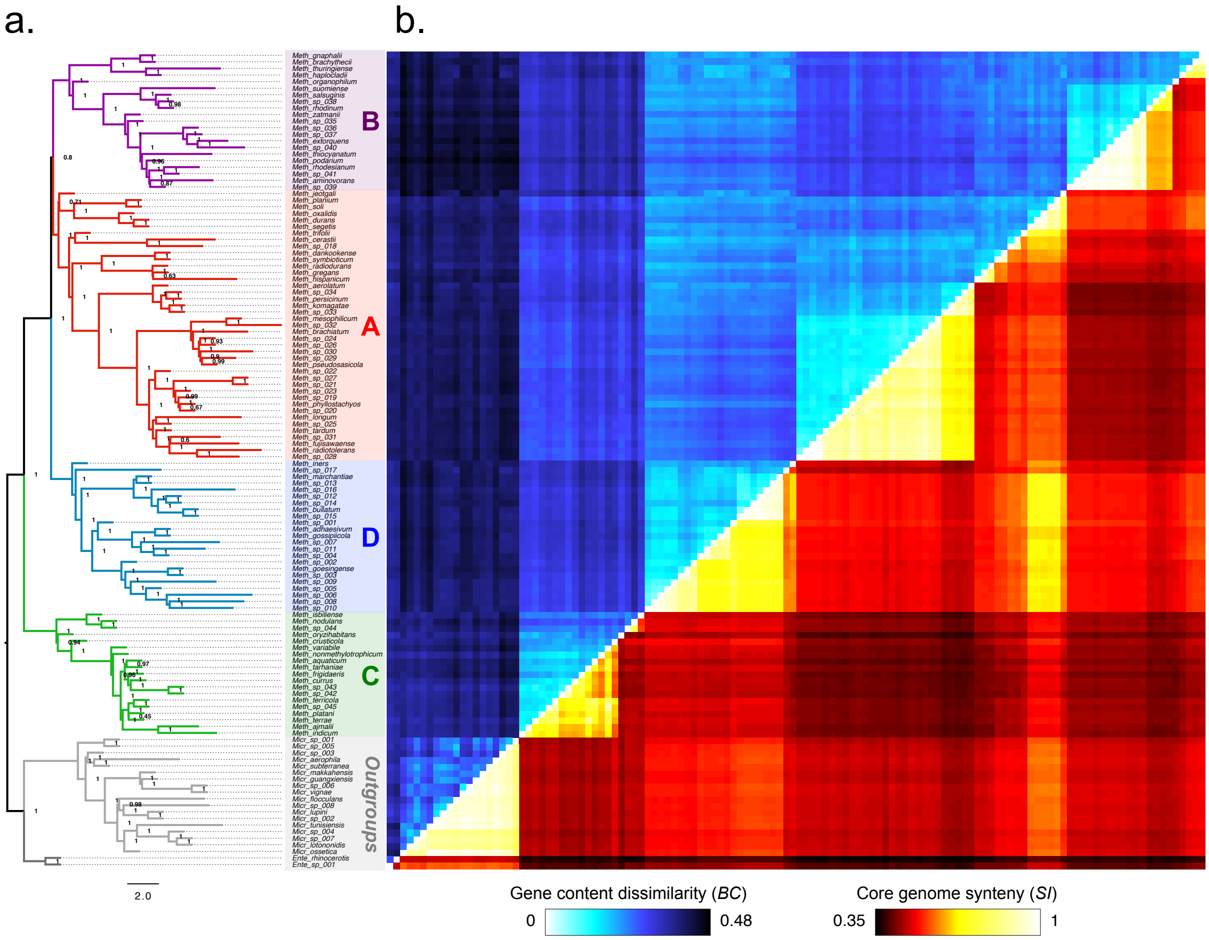

Supplement: evac123_Supplementary_Data [file evac123_supplementary_data.zip › Figure-S8-New.pdf]
