# Supplementary material for: Comprehensive Phylogenomics of Methylobacterium Reveals Four Evolutionary Distinct Groups and Underappreciated Phyllosphere Diversity
Source: Genome Biol Evol. 2022 Jul 30;14(8):evac123. doi: 10.1093/gbe/evac123 (PMC9364378; doi:10.1093/gbe/evac123)

Figure S9: Detailed *Methylobacteriaceae* lineage trees inferred from gene content (a) and core genome synteny (b). Each ML tree was inferred in RAxML assuming a BINCAT model (1,000 replicated trees). Nodal support values indicate the proportion of replicate tree supporting each node. Trees were rooted on *Microvirga* and *Enterovirga*. Branches were colored according to assignation to *Methylobacterium* groups (A: red; B: purple; C: green; D: blue) and outgroups (*Microvirga*: grey; *Enterovirga*: dark grey)

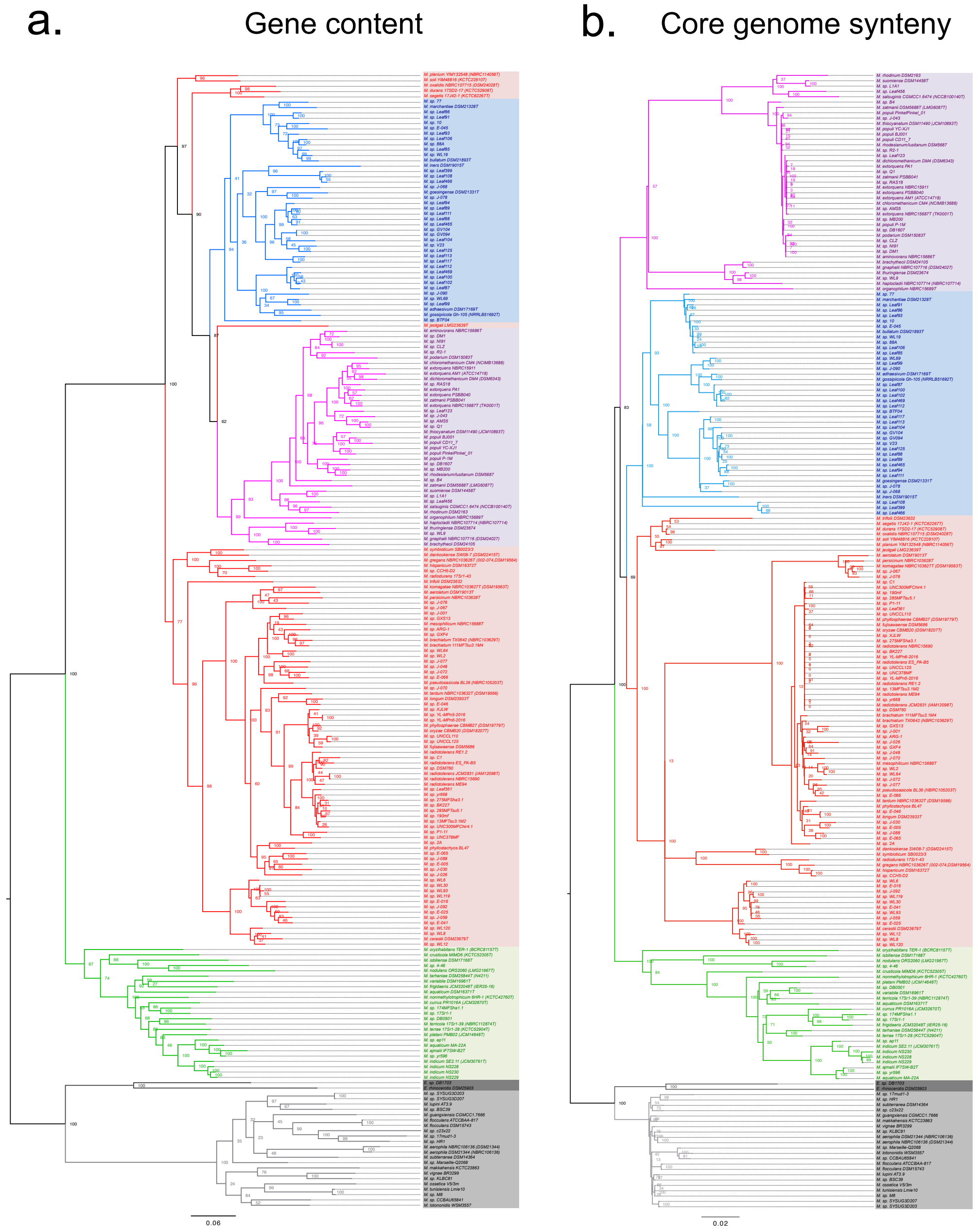

Supplement: evac123_Supplementary_Data [file evac123_supplementary_data.zip › Figure-S9-New.pdf]
